# Supplementary material for: All-atom molecular dynamics of the HBV capsid reveals insights into biological function and cryo-EM resolution limits
Source: eLife. 2018 Apr 27;7:e32478. doi: 10.7554/eLife.32478 (PMC5927769; doi:10.7554/eLife.32478)
Supplement: Transparent reporting form [file elife-32478-transrepform.docx]

***eLife’s* transparent reporting form**

We encourage authors to provide detailed information *within their submission* to facilitate the interpretation and replication of experiments. Authors can upload supporting documentation to indicate the use of appropriate reporting guidelines for health-related research (see [EQUATOR Network](http://www.equator-network.org/%20)), life science research (see the [BioSharing Information Resource](https://biosharing.org/)), or the [ARRIVE guidelines](http://www.plosbiology.org/article/info:doi/10.1371/journal.pbio.1000412) for reporting work involving animal research. Where applicable, authors should refer to any relevant reporting standards documents in this form.

If you have any questions, please consult our Journal Policies and/or contact us: [editorial@elifesciences.org](mailto:editorial@elifesciences.org).

**Sample-size estimation**

- You should state whether an appropriate sample size was computed when the study was being designed
- You should state the statistical method of sample size computation and any required assumptions
- If no explicit power analysis was used, you should describe how you decided what sample (replicate) size (number) to use

Please outline where this information can be found within the submission (e.g., sections or figure legends), or explain why this information doesn’t apply to your submission:

We performed all-atom MD simulations of the complete HBV capsid without symmetry constraints for 1.1 µs. Due to computational expense, this is only the third all-atom simulation of a virus capsid to reach the microsecond timescale, and thus upholds the highest standard in sampling for such a system.

**Replicates**

- You should report how often each experiment was performed
- You should include a definition of biological versus technical replication
- The data obtained should be provided and sufficient information should be provided to indicate the number of independent biological and/or technical replicates
- If you encountered any outliers, you should describe how these were handled
- Criteria for exclusion/inclusion of data should be clearly stated
- High-throughput sequence data should be uploaded before submission, with a private link for reviewers provided (these are available from both GEO and ArrayExpress)

Please outline where this information can be found within the submission (e.g., sections or figure legends), or explain why this information doesn’t apply to your submission:

Due to computational expense, the present study is performed based on a single trajectory capturing 1.1 µs of sampling from MD simulations of the HBV capsid. Data analysis was performed on the final 1.0 µs of sampling.

**Statistical reporting**

- Statistical analysis methods should be described and justified
- Raw data should be presented in figures whenever informative to do so (typically when N per group is less than 10)
- For each experiment, you should identify the statistical tests used, exact values of N, definitions of center, methods of multiple test correction, and dispersion and precision measures (e.g., mean, median, SD, SEM, confidence intervals; and, for the major substantive results, a measure of effect size (e.g., Pearson's r, Cohen's d)
- Report exact p-values wherever possible alongside the summary statistics and 95% confidence intervals. These should be reported for all key questions and not only when the p-value is less than 0.05.

Please outline where this information can be found within the submission (e.g., sections or figure legends), or explain why this information doesn’t apply to your submission:

We performed MD simulations of the HBV capsid for 1.1 µs. Analyses employing statistical averages used a sample size of all 50,000 frames collected during the final 1 µs of simulation; standard deviations are reported or plotted in the Results section of the manuscript.

(For large datasets, or papers with a very large number of statistical tests, you may upload a single table file with tests, Ns, etc., with reference to sections in the manuscript.)

**Group allocation**

- Indicate how samples were allocated into experimental groups (in the case of clinical studies, please specify allocation to treatment method); if randomization was used, please also state if restricted randomization was applied
- Indicate if masking was used during group allocation, data collection and/or data analysis

Please outline where this information can be found within the submission (e.g., sections or figure legends), or explain why this information doesn’t apply to your submission:

Group allocation is not applicable to the present study.

**Additional data files (“source data”)**

- We encourage you to upload relevant additional data files, such as numerical data that are represented as a graph in a figure, or as a summary table
- Where provided, these should be in the most useful format, and they can be uploaded as “Source data” files linked to a main figure or table
- Include model definition files including the full list of parameters used
- Include code used for data analysis (e.g., R, MatLab)
- Avoid stating that data files are “available upon request”

Please indicate the figures or tables for which source data files have been provided:

The source data set for the present study is a 1.1 µs simulation trajectory (55,000 frames) for the complete assembly domain of the HBV capsid in explicit solvent (6 M atoms), the storage footprint for which is 4 TB. Due to sheer size, it is unfeasible for us to upload this data to a standard repository. Nevertheless, the trajectory is currently being hosted by us on local storage resources at the University of Delaware and is available upon request.

Source files are provided in plain text format for the numerical data presented in Figures 1-5.

The code used to obtain these values is accessible as part of freely available software cited in the manuscript, or is planned for publication via a forthcoming manuscript. Algorithms for the latter are clearly described in the Methods section of the manuscript to facilitate reproducibility of results.
